# Parents’ lived experience of adolescents’ repeated non-suicidal self-injury in China: a qualitative study

**DOI:** 10.1186/s12888-022-03715-7

**Published:** 2022-01-28

**Authors:** Xu Wang, Xuehua Huang, Xia Huang, Wenting Zhao

**Affiliations:** 1 Mental Health Center of West China Hospital, Sichuan University, No. 28 South Dianxin Street, Chengdu, 610041 China; 2 West China School of Nursing, Sichuan University, Chengdu, China

**Keywords:** Non-suicidal self-injury, Repeated self-injury, Adolescent, Parent, Qualitative study

## Abstract

**Background and objectives:**

The prevalence of non-suicidal self-injury (NSSI) is high among adolescents. Parents have significant impact on the development of NSSI. Many quantitative studies have demonstrated the relationship between parental factors such as parenting behaviors and adolescents’ NSSI. However, few studies have explored parents’ responses and adolescent-parent reciprocal interaction during repeated NSSI. This study aimed to explore parents’ cognition, behaviors and adolescent-parent reciprocal interaction during repeated NSSI.

**Methods:**

This is a phenomenological study. By purposive sampling, 24 parents of adolescents with repeated NSSI were recruited from a child and adolescent psychiatric ward in a mental health center in Chengdu, China. Semi-structured interviews were conducted and audio-recorded. Audio-recordings were transcribed verbatim and analyzed using thematic analysis.

**Findings:**

Three themes were identified: parents’ attribution, perceptions and coping behaviors of NSSI. Chronic stress of adolescents and triggers of NSSI were associated with parental expectations. Parents initially perceived NSSI as a manifestation of puberty, a way of making needs met or a coping strategy of negative emotions, and gradually realized that it was a condition requiring psychological assistance. Parents’ coping behaviors of NSSI were divided into 4 stages, namely denial, dissuasion, reflection and adaptation, and working as a team. To be more specific, parents’ coping strategies at dissuasion stage included criticizing and conciliating, while those at reflection and adaptation stage included neglecting, avoiding conflicts and increasing control.

**Conclusions:**

These findings suggest that adjusting parents’ expectation and negative perceptions of NSSI is beneficial to reduce adolescent-parent conflict and adolescents’ inner conflict and prevent NSSI. Furthermore, it’s necessary to publicize NSSI related knowledge to promote the early detection and treatment of NSSI.

## Background

Non-suicidal self-injury (NSSI) refers to behaviors that individuals intentionally take to damage their own body tissues without suicidal intent, which are not socially sanctioned, including burning, cutting, stabbing and hitting oneself [1]. NSSI usually starts in early adolescence with an estimated global prevalence of 17.2% in nonclinical samples [2]. NSSI is even more common among adolescents with mental disorders, such as eating disorder, borderline personality disorder and depression [3–5]. NSSI often occurs repeatedly. Multiple episodes of NSSI not only lead to serious health problems such as infection and even autoamputation of body tissues [6, 7], but also generate other issues like increased stress within the family and financial difficulties caused by subsequent medical treatments [8].

There are many theories about why people perform NSSI. Cognitive-Emotional Model [9] explains NSSI at an individual level. It suggests that NSSI is an emotion regulation strategy. Individuals with a belief in which NSSI would help them achieve a desired mental state when presented with an emotionally volatile situation have an increased risk of performing NSSI. Nock [10] took a functional and interpersonal approach, and proposed a Four-Function Model, which believed that NSSI has not only intrapersonal functions like regulating one’s emotion/cognition but also interpersonal functions like communicating with others. In both theoretical models, early life experience, such as childhood maltreatment and familial hostility, contributes to the onset and maintenance of NSSI.

Empirical works have identified such correlates. A systematic review and meta-analysis of 71 publications found that childhood maltreatment and its subtypes (sexual abuse, emotional abuse, physical abuse and neglect) were associated with NSSI [11]. A longitudinal study of 2127 teenage girls revealed that parental harsh punishment and low parental monitoring predicted increased odds of subsequent onset of NSSI, while positive parenting behaviors reduced the odds of next year NSSI onset [12]. The results of an investigation of 957 undergraduate students showed that perceived parent-child relational trauma, including parent alienation and parents’ having abdicated parental roles, was uniquely linked with addictive features of NSSI [13]. In summary, based on previous studies of adolescents with NSSI, parents played important roles in the onset and maintenance of NSSI.

Waals et al. [14] put forward the Family Distress Cascade Theory, which explored the reciprocal interaction between NSSI and adolescent-parent relationship. It proposed that parents exposed to a family member’s NSSI would experience acute stress and chronic secondary stress when they can’t anticipate the occurrence of next NSSI. Such stress leads to subsequent controlling parenting behaviors, which would cause increased episodes of NSSI, as adolescents viewed NSSI as a means of fighting for autonomy while maintaining certain connectedness with parents. This theory explained that how parents’ controlling behaviors led to more frequent and severe NSSI.

However, this may not be the whole picture of adolescent-parent relationship and reciprocal interaction during repeated NSSI. Fu et al. [15] interviewed parents of adolescents who self-injured about the impact NSSI had on them. Most parents reported being more patient and willing to communicate with their children as friends. A three-year prospective study [16] using cross-lagged analysis has confirmed that presence of NSSI in year 1 was significantly associated with an increase in parent-report punishment in year 2, while continuous NSSI in year 2 was strongly related to more supportive parenting behaviors in year 3. It suggested that parents’ responses to NSSI were changing over time. But it was unclear whether there was a pattern of parental behaviors in response to repeated NSSI and how it affects the path of NSSI.

This study further investigated parents’ lived experiences of their children’s repeated NSSI, exploring parents’ cognition and behaviors and adolescent-parent reciprocal interaction during repeated NSSI from a cognitive behavioral approach. According to cognitive behavioral theory, an individual’s cognition has significant influence on the development and maintenance of behaviors associated with specific life events. We hope to provide more theoretical basis for NSSI intervention.

## Methods

### Study design

This qualitative study took a phenomenological approach, which attempts to understand human experience and uncover meanings. This approach allows researchers to rely on participant’s perspectives on the phenomenon, while recognizing the influence of researcher’s own background and experience. Therefore, it is suitable for this study.

### Research setting

The study was conducted from October 2020 to January 2021 at the mental health center of a tertiary hospital in Chengdu, Sichuan province, China. The tertiary hospital we choose is the largest regional medical center in the city. There is a child and adolescent psychiatric ward with 75 beds in the mental health center, where we selected and interviewed our participants. In the ward, a multidisciplinary health care team, including psychiatrists, psychiatric nurses, psychotherapists and occasionally nutritionists, will work with patients and their family members to develop treatment plans. Treatment options include but are not limited to medication, psychotherapy and physical therapy.

### Participants and recruitment

Purposive sampling methods were used. When adolescents with a history of repeated NSSI was admitted in the child and adolescent psychiatric ward, we screened them first. When adolescents were between 12 to 18 year old, had no psychiatric symptoms such as delusion and hallucinations, and started NSSI within 3 years, then we check their parents for inclusion criteria: (1) is the biological parent; (2) can speak and understand at least one dialect around Sichuan or Mandarin; (3) has been with the adolescent from his or her first self-injury to the present; (4) has no cognitive problems and can complete an interview of about an hour; (5) gives informed consent. Diversity of genders, educational level, number of children and long-term residence were considered when recruiting participants. If parents met the inclusion criteria, members of the research team would explain the content of the study and invite them to participate. When informed consent was achieved, a face-to-face interview would be arranged in the ward during hospitalization.

When there was no new relevant knowledge emerging from interviews, we stopped recruiting more participants. In the end, 26 parents who met the study criteria and varied in genders, educational levels, number of children and long-term residence were approached during the process. But 2 parents refused to participate due to concerns about privacy leakage. Twenty-four parents were recruited and accepted a standard, semi-structure interview.

### Research team

Wang (A1) and Huang (A2) are psychiatric nurses who have worked in the mental health center for 8 years and 28 years, respectively. Both of them have the license to practice mental health counseling in mainland China. A1 and A2 were responsible for study design, interviewing participants face-to-face and data analysis. These two authors were not involved in the medical services of adolescents who participated in this study. Participants and authors didn’t know each other before scheduled interviews. Huang (A3) and Zhao (A4) were responsible for recruiting and screening participants and transcribing interviews verbatim since they were familiar with dialects within Sichuan province. All authors have been trained with qualitative research methods.

### Data collection

We conducted 24 one-on-one semi-structured interviews with participants in a psychotherapy room of the child and adolescent psychiatric ward. The interview guide was developed based on our interests of study and research gaps identified from literature review, and was adjusted after analysis of the first five interviews. The main adjustment was to guide parents to recall and narrate clearly with adolescents’ NSSI history as the story line, so as to make their answers more organized. There was no pilot study. Each interview started with a brief introduction to the aim and content of this study, and reassured participant’s permission to audio-record the interview. The interviews used open-ended questions and focused on 5 topics: (1) history of adolescent’s NSSI (onset, maintenance and interventions); (2) situations or patterns of adolescent’s NSSI; (3) participant’s cognition and behaviors when found about adolescent’s NSSI; (4) adolescent’s responses to participant’s responses over time; (5) changes in participant’s cognition and behaviors during repeated NSSI. Examples of question: What was the first time that you can remember the child hurt himself/herself? How did you find out? What did you think were the causes of such behaviors? What was your response? (Prompts: what did you say or do?). Why did you respond in that way? (Prompts: what did you think? how did you feel?) How did he/she react to your response? Has children’s self-injury behavior changed at the beginning compared with now? If so, what changed? Has your response changed? If so, what changed? Each interview lasted 40 to 60 min. No repeat interviews were conducted. Interviews were conducted in Mandarin or Sichuan dialect according to participants’ preference.

### Data analysis

Data analysis began concurrently with the first interview. A3 and A4 transcribed interview recordings verbatim into texts. Transcripts were entered into NVivo 11 [17] for further analysis. Thematic analysis was used, following the six-phased process described by Nowell [18]. It is a rigorous and flexible method that can be used to identify, analyze, organize, describe and report themes found within a data set across a range of epistemologies and research questions [19]. A1 and A2 completed the first three phases separately, including reading and re-reading interview transcripts to familiarize with raw data, generating initial codes by attaching labels to identified interesting aspects in the data, and searching for themes by sorting related codes hierarchically. All researchers reviewed coded data under subthemes to assure a coherent pattern together. Themes and subthemes were compared between cases to determine if they needed to be refined according to their supportive data. Research team members were convened biweekly to discuss results of each phrase before the next phrase began. Research team, together with advisory experts (one psychiatrist and one mental health counselor, both specialized in adolescent’s mental problems, and one nursing professor with abundant qualitative research experience), read through all data, scrutinized coding, defined and redefined themes until the scope and content of each theme could be clearly and succinctly described and consensus was reached.

### Rigor and trustworthiness

Credibility, dependability, confirmability and transferability were used to develop rigor and trustworthiness in this study [20]. Credibility was ensured by in-depth interviews with participants, triangulation (where multiple researchers were involved to provide different perspectives) and peer debriefing. Dependability was achieved by external audit, i.e., each step in the process of data collection and data analysis was examined by advisory experts. Confirmability was reached by reflective journals written by researchers detailing what they did during the study and why. Considering the mental health counseling background of the two authors responsible for interviews, reflective journals were written for audit in order to reduce researcher bias. Transferability was established by purposive sampling and a thick description of data.

## Findings

Table 1 describes demographic characteristics and NSSI related information of adolescents and their parents.

**Table 1:** Demographic characteristics and NSSI related information

Characteristics	Adolescents (***n*** = 24)	Parents (n = 24)
**Gender**		
Female	21	18
Male	3	6
**Age (years) (Mean ± SD)**	15.0 ± 1.7	42.5 ± 5.3
**Months from first NSSI (Mean ± SD)**	16.1 ± 9.0	–
**Education**		
Primary school	–	1
Junior high school	–	12
Senior high school	–	7
Bachelor’s degree	–	4
**Marital status**	–	
Married	–	15
Divorced	–	7
Remarried (one or both)	–	2

Through interviews with parents about their experience of adolescents’ repetitive NSSI, 3 themes and 10 sub-themes were identified (Table 2). (1) “Attribution of NSSI” with the sub-themes: chronic stress resulting from parental expectations; triggers. (2) “Perceptions of NSSI” with the sub-themes: a manifestation of puberty; a way of making needs met; a coping strategy of negative emotions; a condition that requires psychological assistance. (3) “Coping behaviors of NSSI” with the sub-themes: denial; dissuasion; reflection and adaptation; working as a team.

**Table 2:** Themes and sub-themes

Themes	Sub-themes
Attribution of NSSI	Chronic stress resulting from parental expectations
	Triggers
Perceptions of NSSI	A manifestation of puberty
	A way of making needs met
	A coping strategy of negative emotions
	A condition that requires psychological assistance
Coping behaviors of NSSI	Denial
	Dissuasion
	Reflection and adaptation
	Working as a team

### Attribution of NSSI

When asked about adolescent’s first self-injury, parents generally began with a deep review and analysis of adolescent’s past behaviors and life experiences, looking for clues that they thought led to or strongly associated with NSSI. Attribution of NSSI reflected how parents perceived it, and partially explained the way they reacted.

#### Chronic stress resulting from parental expectations

Almost all parents mentioned that there was chronic and persistent stress in the development of their children. The stress came from roles and responsibilities that adolescents were expected to assume. Among them, the two most prominent parental expectations were maintaining good family relationships and obtaining excellent academic performance. However, various factors in reality hindered adolescents from meeting these parental expectations. The contradiction between reality and parental expectation caused fierce and constant inner conflicts of adolescents, forming chronic stress.

*“Her mother and I have busy work schedules and are usually not at home. She is mainly raised by her grandma. However, her grandma has a serious preference for sons, pays most of attention to her little brother, and is not genuinely concerned about her problems.”-P02.(M, 38)*

*“Our relationship (between two families) is special. Her father was brought up by her uncle and aunt. She would be at their place for dinner when we were out for work. But she was reluctant to go. It may be her aunt’s way of talking…She mentioned before, when we were not there, her aunt verbally abused her.”-P08.(F, 46)*

#### Triggers

In most parents’ experiences, there was always a trigger for NSSI. A trigger referred to an event that caused violent mood swings in adolescents. The trigger itself seemed trivial from parent’s perspective, but stimulated adolescents to generate overwhelming negative emotions that were inconsistent with the severity of the event. Self-injuries generally happened immediately after the trigger.

One adolescent had multiple triggers. The trigger for NSSI could be different each time. However, adolescents exhibited persistent over-sensitivity to triggers related to parental expectations, which were also the most common causes of conflicts between adolescents and their parents.

*“She valued her phone above her life……Her father and I decided to take away her phone until her academic performance improved. She was very upset and wanted her phone back. But we were quite determined. There was a fierce quarrel. She cut her wrist right after that.”-P03.(F, 38)*

*“We usually get along very well, but when it comes to study, she is very irritable and easy to lose temper.”-P06.(M, 41)*

### Perceptions of NSSI

There were four main perceptions of NSSI among parents, which appeared in 2 stages of repeated occurrence of NSSI. The first few times that parents found out about adolescent’s NSSI, they regarded it as a manifestation of puberty, a way of making needs met or a coping strategy of negative emotions. But as NSSI happened more frequent or results of NSSI became more severe, parents looked for other possible explanations, and gradually recognized NSSI as a condition that required psychological assistance.

#### A manifestation of puberty

Many parents equated NSSI with puberty when they discovered adolescents’ self-injury behaviors for the first few times. Although parents recognized that self-injury was an abnormal behavior, some even accompanied by negative emotion, they normalized it under the context of puberty.

*“I have noticed that there were some wounds on her hands, not deep. I thought it was just puberty. She told me that some of her friends did that too……”-P08.(F, 46)*

*“I was shocked when her teacher told me about her self-injury at school……And confusing too……Why would she do that? I knew she was upset, but, you know,isn’t is normal kids at her age feel upset??”-P06.(M, 41)*

#### A way of making needs met

Another common perspective parents had on NSSI at first was that, it was a way for adolescents to threaten and force parents to meet their spiritual or material needs. Parents believed that NSSI was a tool, which adolescents used to express their discontent, seek for attention and bargain for autonomy. Such parents and adolescents have frequent conflicts. Some adolescents escalated NSSI when parents refused to accede to their demands.

*“She asked us for allowance and we gave it to her. But she asked for more and more, so we refused. Then she claimed that she was in a bad mood and made quite a scene. ”-P19.(F, 46)*

#### A coping strategy of negative emotions

A few of the interviewed parents perceived that for adolescents, NSSI was not only a way to express bad feelings, but also a strategy of coping with negative emotions. Parents heard from adolescents that NSSI made them feel better, or that physical pain caused by NSSI drove away or relieved their mental pain. As NSSI repeated, some adolescents escalated methods of NSSI and chose those caused more severe physical pain.

*“Her self-injury is, I think, a kind of resistance to mental pain……I knew it was not suicide. Otherwise the cut wouldn’t be so superficial.”-P04.(F, 50)*

*“I asked her, ‘Don’t you feel pain?’ She said, ‘Comfortable. It was painful at the beginning, but comfortable.’ She cut deeper and deeper! She claimed that only deep cut can make her feel better now.”-P01.(F, 42)*

#### A condition that requires psychological assistance

At the early stage of NSSI, parents thought it was abnormal but within control. As NSSI continued, or its frequency and/or severity increased, parents were gradually aware that repeated NSSI was a condition that did not change automatically, nor can it be completely resolved by themselves. As strange as it may sound, in most cases, not until a straightforward message that “these adolescents need psychiatric interventions” was sent to parents, did they recognize that NSSI was a condition that required psychological assistance. Such message came from adolescents’ self-reports, reminders from relatives or friends with related experiences or knowledge, or cases having the same symptoms that parents found online.

*“I knew she hurt herself. But at that time, I didn't know what to do, so I called her aunt. Her aunt is a doctor and a mental health counselor. She told me my daughter needed to see a psychiatrist.”-P24.(F, 45)*

*“I noticed her cuts a year ago, but I was not sure……As she grew older, things didn’t seem to get better. My friend said it might be depression. So I searched on the Internet, and realized it was serious.”-P05.(F, 34)*

### Coping behaviors of NSSI

Through interviews, we found that parents’ coping behaviors of NSSI can be divided into four stages, namely denial, dissuasion, reflection and adaptation, and working as a team. Behind the changes in coping behavior at each stage are the changes in parents’ cognition.

#### Denial

In some cases, adolescents initially tried to conceal the fact that they were intentionally hurting themselves from parents. When parents saw and asked about the scars of self-injuries, they claimed that those were caused by accidents. Parents had doubts, but self-injury challenged their current perceptions of adolescents and adolescent-parent relationship, so they denied such possibility.

*“She said it (cut on the wrist) was an accident. She used to be so afraid of pain and seeing blood. How can she cut herself intentionally?”-P11.(F, 49)*

#### Dissuasion

At this stage, parents ascertained that adolescents engaged in NSSI on purpose and tried to dissuade adolescents from doing it again in two ways. One was to criticize, the other was to conciliate.

Some parents criticized such behaviors and adolescents for doing so in order to prevent it from happening again. They pointed out that NSSI was wrong and expressed their anger.

*“I saw her scars on the wrist, not deep. I immediately told her, “You can’t do this! I am angry that you did that!”.”-P16.(F, 46)*

Some parents stopped adolescents’ self-injuries in a more gentle way. They conciliated adolescents, asking about their feelings and reasons behind NSSI, expressing concerns about possible subsequent health problems and giving advise on how to cope with triggers.

*“I told her that this wouldn’t solve her problems and could lead to serious physical damage. I told her we love her, she can just ask for help.”-P22.(F, 45)*

#### Reflection and adaptation

Most parents reflected their previous words and behaviors, adapted to adolescents’ responses, and then changed coping strategies, when they have failed several times to prevent NSSI by verbal expression. At this stage, the main coping strategies adopted by parents included neglecting, avoiding conflict and increasing control.

When parents have failed to prevent NSSI and didn’t think current self-injury behavior would cause any serious health problem, they neglected it and remained the previous adolescent-parent reciprocal interaction mode. Such parents usually regarded NSSI as adolescents’ way of making needs met, and believed that neglect can help NSSI subside.

*“I thought paying attention was not good for her. Next time she wanted something, she would do it again. So I just let it go.”-P04.(F, 50)*

However, after experiencing adolescent’s repeated NSSI and several failures to stop it, some parents tried their best to avoid conflict scenes and exhibited a certain degree of compromise, such as lowering academic requirements and meeting some of adolescents’ needs.

*“She was defensive, even angry, towards me. Maybe because I was too strict with her in her study. Now that she's like this (cutting the wrists), I don't want to ask for anything anymore.”-P10.(M, 57)*

When parents recognized that adolescents had trouble controlling themselves from engaging in NSSI, they increased controlling parental behaviors, including but not limiting to strengthening supervision, putting away knives and limiting peer companions with whom they thought to be “bad influence”.

*“I was afraid that she might do something stupid. Because she has done it before. So I slept with her all the time.”-P20.(F, 47)*

*“I felt terrible, but I can’t help. When she was not home, I put away the knives stealthily. When she asked, I said I didn't know.That's all I can do......”-P08.(F, 48)*

#### Working as a team

In the process of trying various strategies to deal with NSSI, parents gradually realized that disregarding adolescents’ will to prevent NSSI would not be effective. Only measures that adolescents were willing to cooperate with had positive impact on adolescents’ behaviors. Therefore, parents changed adolescent-parent reciprocal interactions and worked with adolescents as a team, which included adopting better communication strategies, helping with problems raised by adolescents and actively seeking support*.*

*“After that time, I communicated with her. I said she has grown up now, I will change myself. This family can be more democratic, I will listen to her. She can be in charge sometimes.”-P18.(F, 42)*

*“We (parents) are changing too. I used to be tough. When she hurt herself, I would confront her and demand her to tell me why. Now I can put up with it. She doesn’t have to say anything if she doesn’t want to.”-P10.(M, 57)*

## Discussion

This study explored parents’ lived experiences of adolescents’ repeated NSSI and identified 3 themes: attribution of NSSI, perceptions of NSSI and coping behaviors of NSSI. Parents believed that NSSI was the result of chronic stress and triggers associated with parental expectations. Parents’ perceptions of NSSI can be divided into 2 stages. At first, parents regarded NSSI as a manifestation of puberty, a coping strategy of negative emotions or a way of making needs met. Then they realized that NSSI was a condition that required psychological assistance. Similarly, there were 4 stages of parents’ coping behaviors of NSSI, which were denial, dissuasion, reflection and adaptation, and working as a team in sequence.

In terms of attribution, this study showed that parental expectation was the major source of chronic stress for adolescents. Maintaining a good family relationship and achieving excellent academic performance were the most common expectations Chinese parents had for adolescents, in consistent with the emphasis of traditional Chinese culture on such characters [21]. Not only did parental expectations created chronic stress for adolescents, they were also where triggers for NSSI arose. Adolescent-parent conflicts and adolescents’ inner conflicts closely related to unmet parental expectations were most likely to be fixed triggers for NSSI. Such triggers led to acute mental crisis and were associated with adolescents’ psychological characteristics. Low distress tolerance, high impulsivity and emotional reactivity were prospective risk factors for NSSI [22–24]. According to Erikson’s stages of psychosocial development, adolescents need to establish self-identity and be emotionally independent from parents during adolescence [25]. By meeting parental expectations, adolescents obtained positive feedback from parents, which helped form self-identity. But it hindered the task of achieving emotional independence and autonomy [26]. In this sense, conflicts seemed inevitable. However, parental expectations in line with adolescents’ psychosocial development during adolescence may reduce conflicts and thus decrease the occurrence of NSSI.

Based on parents’ perceptions of NSSI, we found two explanations for the escalation of NSSI frequency or severity. First, NSSI was a way for adolescents to cope with conflicts with their parents. Parents who thought NSSI was a way of making needs met generally had frequent conflicts with adolescents. They felt that each conflict was like a battle. When lost a battle, adolescents escalated NSSI behavior in the next one, so that parents feared the consequences of such behavior and adolescents regained control of the situation and obtained an psychological advantage over parents. It was congruent with and further explained results of prior studies that perceived adolescent-parent relational trauma and aggression moved levels of NSSI from moderate to high or maintained high levels [13, 27]. Second, NSSI had an addictive feature. When some adolescents first adopted a NSSI behavior, it effectively neutralize their mental pain. But with the increase of use, NSSI behavior of the same intensity no longer satisfied the need, so NSSI was escalated. This was the addictive feature of NSSI [28]. Adolescents with such addictive feature were at higher risk of lifetime NSSI and needed early professional intervention [29].

Most parents lacked knowledge about NSSI and how it should be treated [15]. It took them some time to realize that NSSI required psychological assistance. However, not all parents put such thought into action. Parents’ prior knowledge of NSSI, access to mental health knowledge and services, and stigma associated with mental disorder significantly affect the actual timing of seeking psychological assistance. Parents who connected NSSI to well-know mental disorders (typically depression) through shared experiences from relatives or friends or online information sought psychological assistance earlier than those who didn’t. In some cases, stigma associated with mental disorder delayed parents in taking adolescents for proper treatment. More than half adolescents with NSSI had not received psychiatric care for this behavior [30]. There is a higher rate of unmet treatment needs with NSSI.

Findings of this study suggested that parents’ perceptions of NSSI did not determine their coping behaviors alone. Parents with the same perception could behave differently. For example, thinking NSSI as a way of making needs met, some parents ignored it, while other parents actively sought help. In contrast, the past adolescent-parent reciprocal interaction can better predict parents’ coping behaviors. Parents used to respond negatively to adolescents’ needs were more likely to deny, criticize, neglect and increase control. Parents used to actively deal with adolescents’ problems were more likely to conciliate, avoid conflicts and work as a team with adolescents.

This study analyzed parents’ cognition and coping behaviors of NSSI and how they affected adolescents’ NSSI through interviews from a cognitive behavioral approach. Previous studies suggested that parents’ behaviors have a significant influence on the development of NSSI. Findings of this study further illustrated that the persistent influence of parents on NSSI may be achieved through the chronic stress of adolescents caused by the conflict between parental expectations and realistic obstacles. Adjusting parents’ cognition and enhancing adolescents’ resilience may help reduce the occurrence of NSSI, but trials are needed to confirm. In addition, previous studies have identified the influence of patents’ coping behaviors on the development of NSSI. This study restored the process of such influence and found that there was a pattern in parents’ coping behaviors. Behavior changes at each stage of this pattern were not only related to parents’ cognition of NSSI, but also related to previous adolescent-parent interaction patterns and information accessibility. Helping parents to recognize that NSSI requires psychological assistance as early as possible and reaching a therapeutic alliance with adolescents are conductive to effective intervention of NSSI.

### Limitations and future work

In spite of the promising findings of this study, there were several limitations. All participants were selected from adolescents with NSSI who had been admitted to hospital, so that the complete experience of parents seeking psychological assistance could be learned. However, more interviews with parents of adolescents with NSSI who have not received any psychological assistance would help to understand what hinders them. In addition, this study explained the development of repeated NSSI from the perspective of parents, and did not explore other elements that could influence adolescent’s NSSI, such as adolescent-parent communication patterns, access to social resources and parent’s coping styles. It is hoped that future studies can make up for the above limitations and provide more theoretical and practical basis for NSSI.

## Conclusions

Through interviews with parents of adolescents with NSSI, we found that parents mainly attributed NSSI to chronic stress and triggers related to parental expectations, parents’ perceptions of NSSI were divided into 2 stages and 4 types, and there were 4 stages of their coping behaviors, which were affected by perceptions of NSSI and adolescent-parent reciprocal interactions. Our findings suggest that parents need to adjust expectations according to adolescents’ psychosocial development to reduce adolescents’ inner conflicts. In addition, changing parents’ negative perception of NSSI to decrease adolescent-parent relational aggression are essential to deescalate conflicts and prevent NSSI from escalating. Adolescents with addictive feature of NSSI have a higher risk of lifetime NSSI and especially need early medical intervention. Strengthen the publicity and popularization of NSSI knowledge, improve parents’ awareness of NSSI and overcome stigma, so as to promote the early detection and treatment of NSSI.

## Data Availability

The datasets analyzed during the current study are available from the corresponding author on reasonable request.
